# PREGNANCY DISORDERS AND MATERNAL CONSEQUENCES: Ethnic disparities in hypertensive disorders of pregnancy

**DOI:** 10.1530/REP-25-0049

**Published:** 2025-05-29

**Authors:** Frances Conti-Ramsden, Antonio de Marvao, Lucy C Chappell

**Affiliations:** ^1^ Department of Women and Children’s Health, School of Life Course Sciences, King’s College London, London, UK; ^2^ British Heart Foundation Centre of Research Excellence, School of Cardiovascular and Metabolic Medicine and Sciences, King’s College London, London, UK; ^3^ Medical Research Council Laboratory of Medical Sciences, Imperial College London, London, UK

**Keywords:** pregnancy, ethnicity, hypertension, pre-eclampsia, ancestry, antihypertensives

## Abstract

**In brief:**

Ethnic disparities in hypertensive disorders of pregnancy (HDP) are well-described but poorly understood, with complex interplays between biological, environmental, socio-cultural and healthcare factors potentially contributing. This article provides a contemporary review of this topic and makes recommendations for research and clinical care to improve outcomes for minoritised women.

**Abstract:**

HDP affect approximately one in ten pregnancies and are associated with increased risk of adverse maternal and perinatal outcomes. Despite advances in prevention of pre-eclampsia and improved management of blood pressure in pregnancy, stark disparities in HDP incidence and outcomes persist across maternal ethnic groups. This article provides a contemporaneous review of the epidemiology of ethnic disparities in HDP, potential contributors to ethnic disparities, and how maternal ethnicity is currently conceptualised and utilised as a risk factor in clinical practice. We present the challenges of utilising ethnicity as a risk factor and suggest actions needed to tackle ethnic disparities in pregnancy hypertension. Women of Black ethnic backgrounds consistently experience a higher risk of pre-eclampsia, HDP and associated adverse outcomes compared to women of other ethnicities across diverse healthcare settings. While traditional cardiovascular risk factors and socioeconomic status contribute to these disparities, they do not fully explain the observed differences. Understanding these disparities requires research examining complex interactions across biological, behavioural, environmental, socio-cultural, and healthcare system factors. Ensuring appropriate diversity in HDP research is crucial for equitable application of incoming genomic and personalised medicine advances. While the fundamental drivers of ethnic disparities in HDP remain to be fully understood, healthcare systems should prioritise optimising blood pressure control during pregnancy and postpartum for women from minoritised ethnic backgrounds. Ensuring minoritised women with lived experience are equal partners in designing and implementing research and initiatives to address these disparities will be critical to their success.

## Introduction

Hypertensive disorders of pregnancy (HDP) are a leading cause of maternal and infant mortality and morbidity worldwide, with a disproportionate burden in low- and middle-income countries (Steegers *et al.* 2010, Say *et al.* 2014, Lawn *et al.* 2016). Affecting approximately 10% of pregnancies globally, significant disparities persist in the incidence and outcomes of HDP across different ethnic groups (Arechvo *et al.* 2022, Jiang *et al.* 2022, Johnson & Louis 2022). Despite advancements in the understanding and management of HDP over recent decades, the drivers of these disparities remain poorly understood.

**Figure 1:**
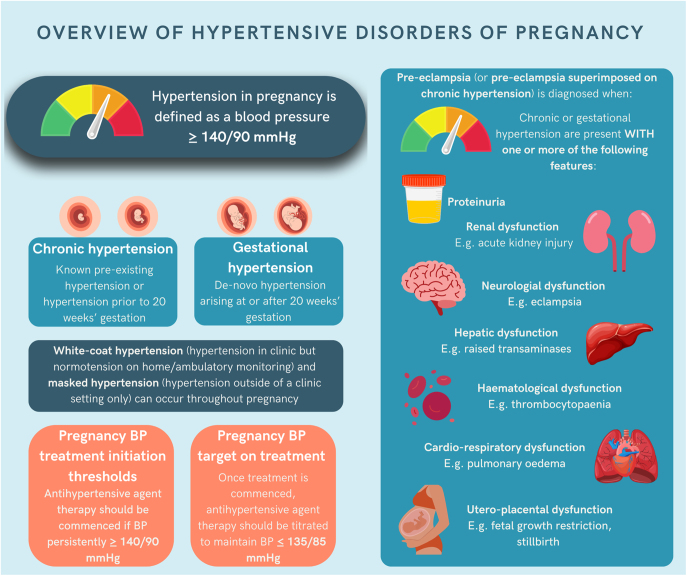
Overview of HDP, including UK NICE guideline recommendations on BP treatment initiation thresholds and BP target on treatments.

HDP encompass a spectrum of conditions including pre-existing chronic hypertension and new-onset hypertensive disorders arising after 20 weeks’ gestation such as gestational hypertension and pre-eclampsia (Fig. 1) (Magee *et al.* 2022*a*). The definition and classification of HDP continues to evolve, particularly for pre-eclampsia, which has broadened from historical criteria of hypertension with proteinuria to modern definitions incorporating maternal organ dysfunction and uteroplacental dysfunction (Magee *et al.* 2022*a*). Whilst optimal definition remains contentious, recent studies suggest broader definitions that can more accurately determine which women and infants are at highest risk of adverse outcomes (Lai *et al.* 2021). While the aetiology underlying HDP, particularly pre-eclampsia, remains incompletely understood, the heterogeneity in clinical manifestations suggests several pathophysiological mechanisms that may lead to a common clinical syndrome recognised by clinicians (Roberts *et al.* 2021).

HDP are well-known to be associated with adverse outcomes including maternal risks of organ dysfunction, stroke, eclampsia and death, and perinatal risks of placental abruption, stillbirth, growth restriction, preterm birth and admission to a neonatal unit (Bramham *et al.* 2014, Say *et al.* 2014, Chappell *et al.* 2021). The past 60 years of research have led to the recognition of blood pressure (BP) thresholds requiring treatment with antihypertensive agents (≥140/90 mmHg) (Tita *et al.* 2022) and establishment of tight BP control targets on treatment (≤135/85 mmHg) to optimise maternal and fetal outcomes including reduction in severe hypertension, pre-eclampsia, placental abruption, preterm birth and neonatal mortality without an increase in small-for-gestational age infants (Magee *et al.* 2015, Tita *et al.* 2022, Attar *et al.* 2023, Chen *et al.* 2023, Conti-Ramsden *et al.* 2024*a*). The UK triennial reports of confidential enquiries into maternal deaths related to pregnancy hypertension have demonstrated that the implementation of a range of initiatives, including free antenatal care, together with national guidelines for antihypertensive and fluid management have been associated with a reduction in deaths in the UK (Conti-Ramsden *et al.* 2019). Initiation of aspirin prophylaxis in high-risk women from the first trimester is also now established as the primary preventative treatment for preterm pre-eclampsia (Rolnik *et al.* 2017). The COVID-19 pandemic has accelerated adoption of out-of-office BP monitoring, enabling earlier diagnosis and remote management (Lavallee *et al.* 2024). However, HDP incidence and maternal outcomes remain starkly different across ethnic groups, with consistently poorer outcomes reported for women from minoritised ethnic backgrounds across diverse healthcare settings (Tanaka *et al.* 2007, Miranda *et al.* 2010, Khalil *et al.* 2013, Urquia *et al.* 2014, Miller *et al.* 2020*b*).

### Scope and methodology of review

This review examines ethnic disparities in HDP, synthesising current evidence on epidemiology and potential contributing factors. We critically evaluate how maternal ethnicity is currently utilized in clinical practice and research, and propose actions to address these disparities. Our methodology involved systematic searches of PubMed and Cochrane databases (January 2020–December 2024) for meta-analyses, systematic and narrative reviews and individual patient data analyses on ethnicity, race, racism and pregnancy hypertension, with snowballing methods and additional searches employed to find additional, relevant literature. Current clinical guidelines from major international bodies (the American College of Obstetricians and Gynaecologists (ACOG) (ACOG 2019, ACOG 2020); the International Society for the Study of Hypertension in Pregnancy (ISSHP) (Magee *et al.* 2022*a*); National Institute for Health and Care Excellence (NICE) (UK) (NICE 2023); Society of Obstetric Medicine of Australia and New Zealand (SOMANZ) (Society of Obstetric Medicine Australia and New Zealand 2023); and Society of Obstetricians and Gynaecologists of Canada (SOGC) (Magee *et al.* 2022*b*) were reviewed to assess the incorporation of ethnicity in current clinical practice. Where evidence gaps exist, we offer perspectives based on expert opinion. Throughout, we follow JAMA guidance for reporting race and ethnicity in medical research (Flanagin *et al.* 2021).

## Ethnicity and ancestry in medical research

Ethnicity is a complex and controversial concept that encompasses shared language, cultural and social traditions, and connotations of common ancestry (Lu *et al.* 2022, Bradby 2003) and can be self-reported or assigned (Johnson & Louis 2022). Modern interpretations of ethnic categories understand these as primarily socio-political constructs with a complex relationship to underlying ancestry (genetics inherited from putative ancestors) (Vyas *et al.* 2020). However, the definition and categorisation of ethnicity vary substantially across countries and continents, with no globally standardized classification system (Bradby 2003, Flanagin *et al.* 2021, Lu *et al.* 2022), and individuals increasingly identifying with more than one ethnic group due to factors such as cultural integration, mixed ancestry, migration, and the growing recognition of complex, multifaceted identities (Bradby 2003, Lu *et al.* 2022). Studies of pregnant women have confirmed the challenge of defining ethnicity in multi-cultural settings, with varying concepts of ethnicity across ethnic groups and a substantial proportion of women feeling unable to describe their ethnicity from available options (Lockie *et al.* 2018). Whilst most medical studies refer to collapsed ethnic groupings (e.g. Alaskan Native or American Indian, Asian, Black, Hispanic, Native Hawaiian or Pacific Islander, Other, and White) (Johnson & Louis 2022), others refer to geographical groupings, e.g. Sub-Saharan African, or more detailed nomenclature (Baiden *et al.* 2024, Conklin *et al.* 2024). In the remainder of this review, we aim to balance consistency in terminology to maximise opportunities for comparison across studies, while incorporating greater granularity where reported as encouraged in JAMA guidelines (Flanagin *et al.* 2021).

As ethnic categories may serve as proxies for primarily social – rather than biological – determinants of health, their inclusion in risk stratification or prediction tools and models is problematic (Vyas *et al.* 2020). Their use often reifies the concept of true biological differences between ethnically defined groups, potentially obscuring the influence of socio-economic, environmental, and structural factors. Consequently, reliance on ethnic categories in clinical tools may not only undermine the precision of medical interventions but also perpetuate systemic inequities in healthcare. As such, calls have been made to remove ethnicity entirely from clinical algorithms (Vyas *et al.* 2020) including pre-eclampsia prediction tools (Sacks & Incerpi 2024), with ethnicity being removed from estimated glomerular filtration rate calculators in 2021 (NICE 2021). The National Academies of Sciences, Engineering and Medicine (NASEM) and the American Journal of Human Genetics have both recently recommended that self-reported ethnicity should not be used as a proxy for genetic ancestral groups, and encourage the reporting and use of genetic ancestry in genomic studies (Feero *et al.* 2024). Previous genomic studies in the US have confirmed that ethnicity is an imperfect proxy for genetically defined ancestry at a population level (Banda *et al.* 2015).

Genetically computed individual ancestry estimates quantifies the proportion of an individual’s genome originating from putative reference ancestral groups (Kumar *et al.* 2010, Mersha & Abebe 2015, Lewis *et al.* 2022). Individuals may not be fully aware of their own ancestry and as modern populations become increasingly genetically admixed, self-allocation of a single ethnic group is likely to be an increasingly unrepresentative proxy for population ancestry. The 1000 Genomes Project has established that, while common genetic variants are shared across continental groups, 86% of rare genetic variants are restricted to one continental group, with the number of shared variants decreasing with geographic distance between populations (Auton *et al.* 2015). In addition, African ancestry individuals show the greatest number of genetic variants in keeping with the Out-of-Africa hypothesis (Auton *et al.* 2015). The historical euro-centric bias in genomics (over 90% of genomic studies are carried out on data from European-ancestry individuals), coupled with high genetic diversity in African-ancestry individuals, has led to calls for large-scale studies of African genomic data to achieve accurate and equitable benefits from genomic studies to all populations globally (Fatumo & Inouye 2023). Small studies have suggested that genetic ancestry is associated with risk of pre-eclampsia independently of maternal ethnicity, suggesting this warrants further investigation (Shahabi *et al.* 2013, Conti-Ramsden *et al.* 2024*b*). Importantly, African populations must benefit equitably from these research findings. Genomics research capability in Africa is growing, spearheaded by the H3Africa initiative (Adebamowo *et al.* 2018), although many challenges to delivery of omic research in LMIC settings remain (Nacis *et al.* 2024).

## Epidemiology of hypertensive disorders of pregnancy across maternal ethnic groups

### The global burden of hypertensive disorders of pregnancy

**Figure 2:**
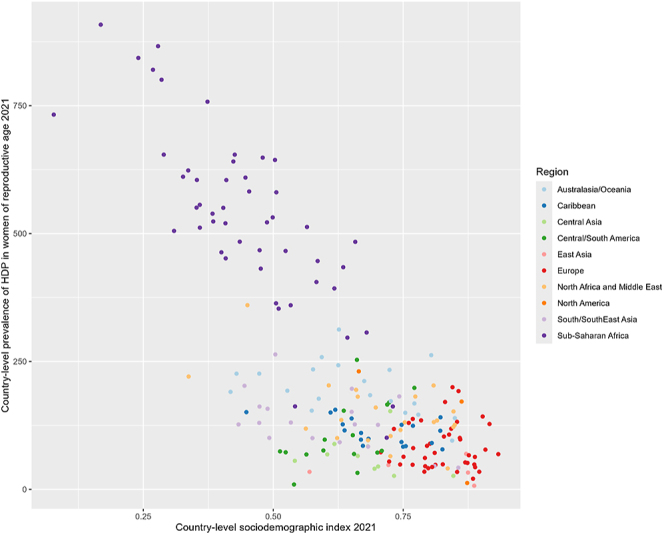
Scatterplot of country-level prevalence of HDP in women of reproductive age (15–49) by country-level socio-demographic index using the 2021 Global Burden of Disease Data (available: https://ghdx.healthdata.org/gbd-2021).

The 2019 Global Burden of Disease (GBD) study data (GBD 2019 Diseases & Injuries Collaborators 2020) estimated the global prevalence of HDP at 116.4 per 100,000 women of reproductive age, with significant disparities by country income (Table 1) (Jiang *et al.* 2022). The highest prevalence was reported in Africa at 334.9 per 100,000, followed by Southeast Asia (136.8), the Eastern Mediterranean (121.4), and North America and the Caribbean (87.4). The lowest prevalence rates were found in South and Central America (45.1) and the Western Pacific region (16.4). The analysis also revealed an inverse relationship between HDP prevalence and both country sociodemographic index (SDI, a representation of country social and economic development) and human development index (HDI, a measure of human development in health, education and standard of living) such that the higher the country SDI or HDI index, the lower the prevalence of HDP (Jiang *et al.* 2022). Plotting of 2021 GBD data demonstrated a very similar relationship (Fig. 2). This trend is corroborated in another epidemiological study utilising GBD data, which additionally noted that while most regions showed declining HDP incidence over time, countries with low SDI and HDI saw increasing rates (Wang *et al.* 2021). Similarly, both disability-adjusted life years (DALYs) and maternal mortality from HDP showed strong negative correlations with country SDI and HDI levels (Jiang *et al.* 2022).

**Table 1:** Prevalence and maternal mortality of HDP by country income category (2019). Data are presented per 100,000 women of childbearing age.

Country category	Prevalence	Maternal mortality
High-income countries	70.3	0.09
Middle-income countries	106	1.2
Low-income countries	286.4	3.1
Global	116.4	1.2

Table adapted from Jiang *et al.* (2022). HDP, hypertensive disorders of pregnancy.

### Pre-eclampsia

Eight reviews have reported on disparities in the epidemiology of pre-eclampsia across maternal ethnic groups in the past five years (Table 2) (Harris *et al.* 2020, Zhang *et al.* 2020*a*,*b*, Fasanya *et al.* 2021, Arechvo *et al.* 2022, Burger *et al.* 2022, Johnson & Louis 2022, Suresh *et al.* 2022). All reported a higher risk of pre-eclampsia in women of Black compared to White ethnic backgrounds (Harris *et al.* 2020, Zhang *et al.* 2020*a*,*b*, Fasanya *et al.* 2021, Arechvo *et al.* 2022, Burger *et al.* 2022, Johnson & Louis 2022, Suresh *et al.* 2022). The only systematic review and meta-analysis published to date (Arechvo *et al.* 2022) reported a pooled, unadjusted risk ratio (RR) for pre-eclampsia in women of Black compared to White ethnic backgrounds of 1.68 (95% CI: 1.48–1.92, 17 studies), but with high study heterogeneity. Notably, only four included studies reported adjusted odds ratios (aOR, all adjusted for maternal age, parity, weight, and smoking as a minimum), with an aOR for pre-eclampsia of 1.90 (95% CI: 1.61–2.23, *n* = 4 studies) for women of Black versus White ethnic backgrounds, with three of the included studies reporting USA data (Gong *et al.* 2012, Ross *et al.* 2019) and one reporting UK data (Arechvo *et al.* 2022). Women of South Asian ethnic backgrounds specifically were also found to be at higher risk of pre-eclampsia in adjusted models only (unadjusted RR: 1.08 (*n* = 8 studies), 95% CI: 0.97–1.21, adjusted OR (aOR): 1.28, 95% CI: 1.19–1.38 (*n* = 4 studies)), with no consistent associations seen for women of East Asian ethnic backgrounds (Arechvo *et al.* 2022). Importantly, the authors highlighted that only two studies included in their meta-analysis were at low risk of bias, and only four studies adjusted for relevant confounders (Arechvo *et al.* 2022).

**Table 2:** Summary of reviews reporting the incidence and/or outcomes of HDP across maternal ethnic groups.

Reference	Methodology	Literature search timeline	Included studies, *n*	Scope
Burger *et al.* (2022)	Systematic search strategy, narrative synthesis	Inception to February 2022	53	All HDP
Arechvo *et al.* (2022)	Systematic review and meta-analysis	Inception to December end 2021	19	Pre-eclampsia only
Suresh *et al.* (2022)	Narrative review	Not stated	Not stated	Pre-eclampsia only
Johnson & Louis (2022)	Narrative review	Not stated	Not stated	Pre-eclampsia only
Fasanya *et al.* (2021)	Narrative review	Not stated	Not stated	Pre-eclampsia only
Harris *et al.* (2020)	Narrative review	2010–2019	30	All HDP
Zhang *et al.* (2020*b*)	Narrative review	2010–2017	Not stated	Pre-eclampsia only
Zhang *et al.* (2020*a*)	Narrative review	Not stated	Not stated	Pre-eclampsia only

HDP, hypertensive disorders of pregnancy.

In a narrative review with a systematic search strategy (Burger *et al.* 2022), most of the 34 included studies comparing pre-eclampsia risk between women of Black versus White ethnic backgrounds reported US data only. A higher risk of pre-eclampsia in women of Black versus White ethnic backgrounds was reported consistently across more than ten US studies (aOR: 1.2–2.3), although study bias and adjustment for confounding were not detailed (Burger *et al.* 2022). The largest US study utilising national inpatient sample (NIS) data reported that women of Black ethnic backgrounds experienced pre-eclampsia in 69.8/1,000 deliveries compared to 43.3/1,000 deliveries and 46.8/1,000 deliveries for women of White and Hispanic backgrounds, respectively (Fingar *et al.* 2017).

Most studies reviewed also reported increased risk of pre-eclampsia in women of Sub-Saharan African origin living in European countries compared to majority White populations, with contributing data from Spain, France, Finland and Denmark (adjusted OR/RR: 1.7–2.5) (Burger *et al.* 2022). Immigration studies comparing pre-eclampsia incidence in foreign-born and US-born women of Black ethnic backgrounds have yielded inconsistent results (Gong *et al.* 2012, Boakye *et al.* 2021). However, a large population based study utilising ICD codes across six countries (Australia, Canada, Denmark, Spain, Sweden and the USA) reported that migrant women from Sub-Saharan Africa had higher risk of pre-eclampsia than native women in these six settings, although the magnitude of risk was modified by the country to which they had migrated (Urquia *et al.* 2014).

Several of the reviews reported lower risk of pre-eclampsia in women of East Asian ethnic backgrounds (aOR: 0.6–0.9) (Zhang *et al.* 2020*b*, Burger *et al.* 2022), with most reporting higher risk of pre-eclampsia in women of Hispanic, American Indian, Alaskan native, South central Asian, Filipino and Hawaiian ethnic backgrounds compared to women of White ethnic backgrounds across several geographical settings (Zhang *et al.* 2020*b*, Fasanya *et al.* 2021, Burger *et al.* 2022, Johnson & Louis 2022).

In a large UK cohort study (*n* = 168,966 pregnancies), women of Black and South Asian ethnic backgrounds were also at higher risk of preterm and early-onset pre-eclampsia compared to women of White ethnic backgrounds in adjusted models (Arechvo *et al.* 2022), as reported in some previous studies (Poon *et al.* 2010, Lisonkova & Joseph 2013).

### Gestational and chronic hypertension in pregnancy

Only one review reported disparities in gestational hypertension, summarising the results of nine published studies (Burger *et al.* 2022). A lower incidence of gestational hypertension was observed in women of Black, Hispanic, Asian/Pacific Islander or North African/Middle Eastern backgrounds living in Europe, the US and South Africa compared to women of White ethnic backgrounds (Burger *et al.* 2022). However, the quality of included studies was not assessed or reported. Only three of the included studies were published in the past decade, reporting population level data from Finland, the US and Norway (Ghosh *et al.* 2014, Sole *et al.* 2018, Bastola *et al.* 2022). All reported lower incidence of gestational hypertension in women of Black or Sub-Saharan African backgrounds, with two additionally reporting higher risk of pre-eclampsia in comparison to the White majority population group (Ghosh *et al.* 2014, Bastola *et al.* 2022).

Similarly, only one review reported disparities in chronic hypertension in pregnancy, summarising the results of 16 studies, of which 11 reported US data examining disparities between women of Black and White ethnic backgrounds (Burger *et al.* 2022). Almost all US studies reported a higher incidence of chronic hypertension in women of Black compared to White ethnic backgrounds, with the risk being 1.4–2.3-fold higher, with one study demonstrating that incidence of chronic hypertension was not affected by the duration of residence in the US (Boakye *et al.* 2021). Similar risks have been reported from studies in the UK (unadjusted OR: 3.0) (Panaitescu *et al.* 2017). The review also highlighted generally lower rates of chronic hypertension in women of Hispanic and Asian/Pacific Islander backgrounds compared to White women living in the US, with the exception of women of Filipino and Samoan backgrounds, and higher rates of chronic hypertension in women of American Indian/American native backgrounds (Burger *et al.* 2022). UK data have also demonstrated higher incidence of chronic hypertension in women of South Asian compared to White ethnic backgrounds (unadjusted OR: 1.93) (Panaitescu *et al.* 2017).

### Short-term outcomes of HDP

All identified reviews that included HDP outcomes reported that women of Black versus White ethnic backgrounds are at the highest risk of adverse outcomes in HDP (Zhang *et al.* 2020*a*, Fasanya *et al.* 2021, Burger *et al.* 2022, Johnson & Louis 2022, Suresh *et al.* 2022). This varies from higher risk of development of severe hypertension and requiring more antihypertensive treatment (Goodwin & Mercer 2005, Johnson & Louis 2022, Suresh *et al.* 2022) to increased risk of severe maternal mortality and morbidity, including eclampsia, stroke, acute heart failure, pulmonary oedema, and acute renal failure (Baiden *et al.* 2024, Minhas *et al.* 2021, Burger *et al.* 2022, Johnson & Louis 2022, Suresh *et al.* 2022). Disparities extend to neonatal outcomes, with infants born to women of Black compared to White ethnic backgrounds, with HDP having a higher incidence of perinatal death, neonatal morbidity, low birth weight and preterm birth (Webster *et al.* 2019, Burger *et al.* 2022, Suresh *et al.* 2022). Most of these studies are from high-income settings. Long-term cardiovascular outcomes are outside the scope of this review, but similar ethnic disparities have been reported in the literature (Suresh *et al.* 2022).

### Epidemiology summary

Epidemiological studies consistently demonstrate a higher prevalence of pre-eclampsia and chronic hypertension in women of Black compared to White ethnic backgrounds, primarily from studies from the US and Europe. Meta-analysed data for pre-eclampsia demonstrate that this association is independent of several confounding traditional medical risk factors. While this aligns with data suggesting higher prevalence of HDP in African regions and amongst some immigrant populations (although high-quality confirmatory studies in these settings are required), there are challenges in accurately characterising ethnic disparities in the literature. Broad ethnic categories commonly used in medical research (e.g. ‘Asian’ or ‘Black’) can mask substantial heterogeneity between subgroups from different geographic or cultural origins. For example, relative risks of HDP have been reported to be very high in women of Filipino backgrounds, but low in women of East Asian backgrounds, despite both being categorised as ‘Asian’ in both UK and US population statistics. In addition, immigration studies reveal that women of the same ethnic background can have different HDP risks based on their country of immigration, highlighting how regional prevalence reflects complex interactions between socioeconomic factors, healthcare access, and environmental exposures that cannot be reduced to ethnic categories alone. Global epidemiological data demonstrate strong inverse trends between country income, sociodemographic and human development indices and both prevalence and outcomes from HDP, further highlighting the importance of social determinants of health and healthcare access in the burden of HDP.

## Potential contributors to ethnic disparities in hypertensive disorders of pregnancy

Seven identified reviews (Table 3) examining contributors to ethnic disparities in HDP discussed four key themes: social and environmental determinants of health, traditional risk factors and comorbidities, race/racism and healthcare systems, and biological/molecular mechanisms (Fasanya *et al.* 2021, Johnson & Louis 2022, Suresh *et al.* 2022, Baiden *et al.* 2024, Conklin *et al.* 2024).

**Table 3:** Summary of reviews investigating aetiology of ethnic disparities in HDP.

Reference	Methodology	Literature search timeline	Included studies, *n*	Scope
Conklin *et al.* (2024)	Narrative review	NS	NS	Pre-eclampsia only
Baiden *et al.* (2024)	Systematic scoping review	01/2000–07/2021	36	All HDP
Suresh *et al.* (2022)	Narrative review	NS	NS	Pre-eclampsia only
Johnson & Louis (2022)	Narrative review	NS	NS	Pre-eclampsia only
Fasanya *et al.* (2021)	Narrative review	NS	NS	Pre-eclampsia only
Zhang *et al.* (2020*b*)	Narrative review	2010–2017	NS	Pre-eclampsia only
Zhang *et al.* (2020*a*)	Narrative review	NS	NS	Pre-eclampsia only

NS, not stated; HDP, hypertensive disorders of pregnancy.

### Social and environmental determinants of health

Social determinants of health (SDoH) are defined by the World Health Organisation as ‘the non-medical factors that influence health outcomes. They are the conditions in which people are born, grow, work, live, and age, and the wider set of forces and systems shaping the conditions of daily life’ (WHO 2024). Socio-economic status (SES), a commonly studied SDoH, has been consistently associated with the risk of pre-eclampsia across reviews (Baiden *et al.* 2024, Conklin *et al.* 2024). Low SES, social isolation, precarious employment and poor housing have also been associated with severity of pre-eclampsia (Baiden *et al.* 2024). Women of Black versus White ethnic backgrounds are more likely to have low SES in the US (Baiden *et al.* 2024). Notably, whilst higher SES and education level is protective against pre-eclampsia and preterm birth in women of White ethnic backgrounds, higher SES and education status does not translate into reduced pregnancy risks for individuals of Black ethnic backgrounds in the US (Fasanya *et al.* 2021, Conklin *et al.* 2024). Several large studies have also demonstrated ethnic disparities in incidence and outcomes of pre-eclampsia remain after adjustment for SES in the US and the UK (Khalil *et al.* 2013, Webster *et al.* 2019, Suresh *et al.* 2022). This highlights the variation in impact of SDoH across ethnic groups and likely multiple disadvantage faced by women of Black ethnic backgrounds. Furthermore, SES are only one measure of SDoH, and therefore, the full causal role of SDoH on HDP incidence and outcomes remains difficult to determine, given the breadth of potential factors and challenges in appropriate representation and quantification in studies (Baiden *et al.* 2024, Conklin *et al.* 2024). A study of life course deprivation with HDP showed a strong association, but the analysis was unadjusted for other confounding variables (Francis *et al.* 2024). Currently, only US Preventative Services Task Force recommendations recognise low SES as a risk factor for pre-eclampsia (Davidson *et al.* 2021).

Environmental exposures, which often co-associate with deprivation, must also be considered. Preliminary studies are suggestive that air pollution may increase pre-eclampsia risk (Zhang *et al.* 2020*b*, Conklin *et al.* 2024) and Black women living in urban areas in the US appear to have higher incidence of HDP than those in the rural areas (Baiden *et al.* 2024). In addition, a meta-analysis has shown a strong independent association between blood lead concentrations and pre-eclampsia risk (Poropat *et al.* 2018). In the US, children of Black ethnic backgrounds have the highest blood lead concentrations compared to those of White and other ethnic backgrounds (White *et al.* 2016), with evidence of vertical lead transmission (Cassidy-Bushrow *et al.* 2017), suggesting that further investigation is warranted, although differences in blood lead levels across ethnic groups have not been demonstrated in UK pregnancy cohort studies (Taylor *et al.* 2013). Food security and food quality is another important environmental factor to consider (Conklin *et al.* 2024). Evidence suggests that diet may affect pre-eclampsia risk (Minhas *et al.* 2022), with a randomised clinical trial demonstrating a Mediterranean diet reduced incidence of small gestational age and a composite secondary outcome including pre-eclampsia (Crovetto *et al.* 2021). Given women of ethnic minorities are at highest risk of food insecurity and poor diet, further studies are warranted (Conklin *et al.* 2024).

### Traditional risk factors and comorbid conditions

As discussed in the section ‘epidemiology section: gestational and chronic hypertension in pregnancy’, women of Black ethnic backgrounds are more likely to have pre-existing chronic hypertension compared to women of White ethnic backgrounds, and have higher prevalence of obesity, diabetes and systemic lupus erythematosus in comparison to other ethnic groups (Zhang *et al.* 2020*a*, Suresh *et al.* 2022). However, ethnic disparities in the incidence and outcomes of HDP remain in large studies adjusting for these well-established, traditional cardiovascular risk factors (Khalil *et al.* 2013, Arechvo *et al.* 2022). Therefore, whilst these factors undoubtedly contribute to the elevated risks of pre-eclampsia in women of Black and South Asian ethnic backgrounds, they are not explanatory in isolation.

### Race, racism and healthcare factors

Racial or ethnic group is increasingly understood to be an important proxy for life experience and racism (Johnson & Louis 2022). Racism can be conceptualised in three domains; structural, individual and internalised, with all types being linked to health outcomes (Flanagin *et al.* 2021, Johnson & Louis 2022). Several authors have hypothesised that ethnicity-related differences in hypertension are the outcome of chronic stress from persistent racial discrimination (Johnson & Louis 2022). Both SDoH health and lifetime experience of racism could affect HDP incidence through chronic stress leading to pro-inflammatory signalling (Conklin *et al.* 2024). Decades of research have established that psychosocial stress dysregulates immune function in pregnancy (Hantsoo *et al.* 2019), and a meta-analysis has reported an association between mental and work stress and HDP (Zhang *et al.* 2013). A pilot study of 49 Black pregnant women demonstrated that women with higher reported social support had lower concentrations of pro-inflammatory cytokines, which could affect pre-eclampsia risk (Giurgescu *et al.* 2015), but larger studies have shown no association between self-reported psychosocial stress and perceived racism with HDP in adjusted analyses (Grobman *et al.* 2018, Lee *et al.* 2024).

Studies on the relationship between ethnicity, access to and quality of antenatal care and HDP are scarce (Suresh *et al.* 2022, Conklin *et al.* 2024). US data have demonstrated that hospitals that care for majority Black populations have poorer outcomes even after adjustment for baseline factors, which may reflect lower healthcare resources in hospital systems serving minoritised populations in an insurance-based system (Suresh *et al.* 2022). However, ethnic disparities in HDP remain in the UK, where the National Health Service (NHS) provides healthcare free at the point of access and promotes guideline-based standards of care in maternity services (Khalil *et al.* 2013, Webster *et al.* 2019). Whilst data on HDP outcomes are yet to be described, physician–patient ethnic discordance has been associated with neonatal mortality and county-level racial prejudice in the US is associated with magnitude of disparities in infant birthweight and preterm birth between women of Black and White ethnic backgrounds (Suresh *et al.* 2022). Racism and bias are also likely to have bidirectional influences. In addition to psychosocial stress impacting health, poor healthcare experiences and mistrust in both health institutions and healthcare professionals mean women of ethnic minority backgrounds may be less likely to seek and utilise healthcare services (Baiden *et al.* 2024, Conklin *et al.* 2024). For example, a study of 872 individuals has found that, whilst women of Black ethnic backgrounds are as likely as White women to be prescribed aspirin, they were less likely to report uptake of aspirin (Ragunanthan *et al.* 2024).

### Biological and molecular mechanisms

#### Serum and placental biomarkers

Angiogenic pathways are now well-established as one of the mechanisms involved in the development of pre-eclampsia, with maternal serum placental growth factor (PlGF) concentrations having additive clinical utility for the first trimester screening of pre-eclampsia, and PlGF and/or soluble fms-like tyrosine kinase-1 (sflt1) having strong performance for diagnostic testing (Chappell *et al.* 2021). However, several reviews highlighted that normal ranges of angiogenic markers across maternal ethnic groups warrant further characterisation (Zhang *et al.* 2020*a*,*b*, Fasanya *et al.* 2021, Conklin *et al.* 2024). A UK study of 29,000 pregnancies published in 2023 found that women of Black, South Asian, East Asian and mixed ethnic backgrounds had higher mean PlGF concentrations than White women, affecting the performance of pre-eclampsia prediction models incorporating these biomarkers across maternal ethnic groups at 35–36 weeks’ gestation (Wright *et al.* 2023). Interestingly, a study of 398 women in Accra, Ghana, demonstrated that both PlGF and PAPP-A multiple of the mean (MoM) values were substantially higher than reference standards in White populations (Browne *et al.* 2016). Whilst the causality underlying these findings is uncertain, the authors postulate increased placental production, lower target site affinity or reduced clearance as potential mechanisms (Browne *et al.* 2016). However, it remains unclear whether these differences in blood concentrations translate into substantially altered clinical test performance, and the challenges of incorporating ethnicity into test performance models remain.

Assessment of angiogenic imbalance is increasingly recommended in international guidelines (Magee *et al.* 2022*a*), with PlGF-based diagnostic tests now recommended in UK NICE diagnostics guidelines for the assessment of women with suspected pre-eclampsia (NICE 2022). As such, performance of angiogenic markers for screening and diagnostic testing across maternal ethnic groups should be further investigated. Given the limitations of ethnicity as a proxy for biological differences between individuals (see section ‘Ethnicity and ancestry in medical research’), further studies are also required to disentangle for which factors maternal ethnic background may be acting as a proxy in determining PlGF and PAPP-A concentrations.

#### Genomics

Heritability studies have long suggested a strong inherited component to pre-eclampsia, particularly pre-term pre-eclampsia (Salonen Ros *et al.* 2000). Candidate gene approaches have highlighted multiple loci potentially implicated in pre-eclampsia pathogenesis highlighted in several reviews (Conklin *et al.* 2024). However, these studies suffer from small sample size and lack of validation. Genome-wide association studies (GWAS) have become the gold-standard for genetic association testing. However, adequately powered genomic studies for pre-eclampsia are lacking. While GWAS for coronary artery disease have included over 250,000 cases (Honigberg *et al.* 2023), the largest pre-eclampsia GWAS to date included just over 20,000 cases (Tcheandjieu *et al.* 2022). Despite the modest sample size, the loci identified in the pre-eclampsia GWAS meta-analysis have highlighted the role of natriuretic peptide signalling, angiogenesis, renal glomerular function, trophoblast development and immune dysregulation (Honigberg *et al.* 2023). As identification of loci associated with disease is almost exponentially proportional to the number of cases and controls included in the GWAS samples, increasing GWAS sample size in pre-eclampsia should drive greater molecular insights. However, the latest pre-eclampsia GWAS discovery analysis included just 20 cases in women of African ancestry (Honigberg *et al.* 2023). As such, currently the individuals most affected by HDP are least likely to benefit from insights gained from genetic studies completed to date.

There is growing interest in *APOL1* genetic variants as a potential high-risk biological pathway to pre-eclampsia in women of West African ancestry (Osafo *et al.* 2022). High-risk variants (G1 and G2) in the gene encoding apolipoprotein L1 (*APOL1*) are common in individuals of West African ancestry but rare in individuals of other ancestry groups. *APOL1* variants account for much of the increased risk of non-diabetic chronic kidney disease and end-stage renal disease in West African populations (Tayo *et al.* 2013, Ulasi *et al.* 2013) and in African Americans (Genovese *et al.* 2010, Tzur *et al.* 2010). Furthermore, *APOL1* is abundantly transcribed in the placenta, autoantibodies against *APOL1* have been demonstrated in the blood of women with preeclampsia, and *APOL1* transgenic mice develop a pre-eclamptic phenotype, increasing biological plausibility of an association (Wen *et al.* 2013, Elliott *et al.* 2014, Bruggeman *et al.* 2016). As pre-eclampsia is an endothelial disease, involving microangiopathy in the glomerulus of the kidney, taken together, these observations suggest that *APOL1* expression in the placenta may play a causal role in pre-eclampsia, which may be influenced by both maternal and infant *APOL1* genotype. Several small studies in the USA and South Africa have suggested an association between maternal and infant *APOL1* variants and pre-eclampsia (Reidy *et al.* 2018, Miller *et al.* 2020*a*, Thakoordeen-Reddy *et al.* 2020, Hong *et al.* 2021). However, larger genetic studies of mothers and their infants with assessment of interaction of effects across the maternal–fetal dyad are required to validate these findings.

### Potential contributions to ethnic disparities in HDP summary

Ethnic disparities in HDP are likely to arise from a complex interplay of multiple factors. Evidence from systematic reviews identifies four potential contributing domains: social and environmental determinants of health, traditional cardiovascular risk factors, racism and healthcare system factors, and biological mechanisms. While socioeconomic status correlates with HDP risk, its protective effects vary across ethnic groups, and ethnic disparities persist after adjusting for traditional cardiovascular risk factors such as chronic hypertension and obesity. Recent research has highlighted ethnic variations in angiogenic markers such as PlGF and genetic factors such as APOL1 variants in West African populations, which warrant further investigation. Environmental exposures, chronic stress from racial discrimination, and healthcare system factors may also contribute to ethnic disparities, although their relative importance currently remains unclear. Understanding ethnic disparities requires a comprehensive multilevel approach that considers the complex interactions between social and environmental factors and healthcare systems with appropriate representation of women of ethnic minority groups in mechanistic (e.g. genomic and proteomic) research.

## Use of maternal ethnicity in current pregnancy hypertension clinical practice

Given the epidemiology and limitations in research investigating aetiology of ethnic disparities in HDP to date, we further investigated how maternal ethnicity is currently conceptualised and utilised in contemporaneous clinical practice. There is a limited role for the use of paternal ethnicity, given the potential for false paternity (when a child is identified as being biologically fathered by someone other than the man who believes he is the father), which has been estimated to affect between 1 and 30% of pregnancies (Bellis *et al.* 2005).

### Screening and prediction of pre-eclampsia

Initiation of aspirin prophylaxis in high-risk women in the first trimester, ideally by 16 weeks’ gestation, is established as a preventative treatment for pre-eclampsia, particularly pre-term pre-eclampsia (Rolnik *et al.* 2017). Therefore, prediction of pre-eclampsia risk in early pregnancy to offer aspirin prophylaxis to high-risk women forms an essential part of early antenatal care. A recent review of the screening of pre-eclampsia is available elsewhere (Chappell *et al.* 2021), highlighting that although performance of screening tools using clinical risk factors or biomarkers alone is modest, most multi-variable pre-eclampsia prediction tools (encompassing combinations of clinical risk factors, biophysical, ultrasound and biomarkers) proposed in the literature lack external and independent validation (Townsend *et al.* 2019). The Fetal Medicine Foundation (FMF) pre-eclampsia prediction algorithm (O’Gorman *et al.* 2016, Wright *et al.* 2019, 2020) combines maternal clinical risk factors (such as maternal age, chronic hypertension, diabetes, chronic kidney disease and *in vitro* fertilisation) with ultrasound and biochemical parameters, and has had the most extensive external validation (16 studies across a range of settings) (Tiruneh *et al.* 2024) of many proposed multi-variable pre-eclampsia models (Tan *et al.* 2018, Townsend *et al.* 2019).

Of the HDP guidelines reviewed (ISSHP (Magee *et al.* 2022*a*); ACOG (ACOG 2019); SOMANZ (Society of Obstetric Medicine Australia and New Zealand 2023); SOGC (Magee *et al.* 2022*b*); and NICE (NICE 2023)), NICE and US guidelines recommend clinical risk factor-based screening only, with all other guidelines (ISSHP, SOMANZ, and SOGC) recommending clinical risk factor-based screening as a minimum but endorsing multi-variable pre-eclampsia prediction tools (such as the FMF pre-eclampsia prediction algorithm), where resources allow (Table 4). Black maternal ethnic background (or related categories such as African American) is identified as an independent risk factor for pre-eclampsia in clinical risk factor-based approaches to pre-eclampsia screening in ACOG guidelines only (ACOG 2020). Notably, this recommendation has been the subject of recent debate (Sacks & Incerpi 2024), with the most recent US Preventative services task force recommendation statement on aspirin use for prevention of pre-eclampsia highlighting that Black ethnic background remains included as a moderate risk factor for pre-eclampsia as a proxy for racism rather than biological propensity to disease (Davidson *et al.* 2021). However, maternal ethnic background is also included in the FMF pre-eclampsia prediction algorithm (O’Gorman *et al.* 2016, Wright *et al.* 2019, 2020). The FMF algorithm, trained on a large, inner-city, multi-ethnic population of pregnant women in UK, London, encodes higher risk of pre-eclampsia for women of Black and South Asian compared to White ethnic backgrounds when calculating baseline (prior) risk (Tan *et al.* 2018, Townsend *et al.* 2019). The impact of the inclusion of ethnicity in guidelines on clinical care and outcomes has not been formally investigated, although comparison of the impact of a clinical risk factor approach to pre-eclampsia screening (NICE guidelines, which does not include ethnicity) versus the FMF algorithm (which includes maternal ethnicity in risk prediction algorithm) on pre-term birth is the subject of the ongoing STARshiP trial (NIHR152762).

**Table 4:** Summary of ethnicity-related recommendations in international hypertensive disorder of pregnancy guidelines.

	AHP prescription	Risk prediction
ISSHP 2021*	None	Ethnicity not listed as a risk factor for pre-eclampsia. Fetal Medicine Foundation pre-eclampsia screening algorithm conditionally recommended
ACOG 2020^†^	None	African-American race listed as a moderate risk factor for pre-eclampsia
NICE 2019^‡^	None	Ethnicity not listed as a risk factor for pre-eclampsia
SOMANZ 2023^§^	None	Ethnicity not listed as a risk factor for pre-eclampsia. Fetal Medicine Foundation pre-eclampsia screening algorithm conditionally recommended
SOGC 2022^║^	None	Ethnicity not listed as a risk factor for pre-eclampsia. Fetal Medicine Foundation pre-eclampsia screening algorithm conditionally recommended

* Magee *et al.* (2022*a*).

^†^ ACOG 2020.

^‡^ Nice Guideline NG133 (2023).

^§^ Society of Obstetric Medicine Australia and New Zealand 2023.

^║^ Magee *et al.* (2022*b*).

### Antihypertensive agents

Despite high-quality evidence that control of hypertension in pregnancy to BP < 135/85 mmHg is beneficial to the woman and fetus (Magee *et al.* 2015, Tita *et al.* 2022), considerable uncertainty regarding the risks and benefits of contemporaneous antihypertensive agent therapy in pregnancy remains. There are little data on the response to antihypertensives in pregnancy stratified by ethnicity (Webster *et al.* 2017), with optimal antihypertensive agent therapy the subject of an ongoing randomised-controlled trial (Ashworth *et al.* 2023, Conti-Ramsden *et al.* 2024*a*). Due to this uncertainty, clinical HDP guidelines make varying recommendations, with labetalol, nifedipine, methyldopa and oxprenolol being the most commonly recommended agents (ACOG 2019, Ashworth *et al.* 2022, Magee *et al.* 2022*a*,*b*, NICE 2023, Society of Obstetric Medicine Australia and New Zealand 2023). Treatment of hypertension in pregnancy with antihypertensive agents has recently been reviewed elsewhere (Conti-Ramsden *et al.* 2024*a*). Notably, substantial variation in first- and second-line antihypertensive agent prescribing exists in clinical practice (Whybrow *et al.* 2020). None of the reviewed HDP guidelines make any recommendations on ethnicity or race-based prescribing of antihypertensive agents.

However, observed variation in prescribing of antihypertensive agents may reflect ethnicity-based prescribing in line with out-of-pregnancy guidelines, with both UK and US chronic hypertension guidelines currently recommending stratification of first-line antihypertensive agents by ethnicity (by black African or African–Caribbean family origin) (NICE 2019, Whelton *et al.* 2018) reflecting proposed differences in hypertension aetiology including response to antihypertensive agents at a population level. Specifically, the guidelines endorse the view that there is a higher prevalence of low-renin hypertension in individuals of African ancestry or Black ethnic backgrounds (Materson 2007, Gupta *et al.* 2010), attenuating response to beta-blockers and angiotensin-converting enzyme inhibitors (which work primarily by suppressing the renin–angiotensin system) and increasing the likelihood of response to calcium channel blockers (which work primarily by vasodilation).

Small studies have been conducted in pregnancy. In a study of 117 pregnant women with treated chronic hypertension, women of Black ethnic backgrounds had lower renin and aldosterone concentrations across gestation (Webster *et al.* 2018). Black maternal ethnic group has been independently associated with the prediction of response to labetalol alongside baseline heart rate and stroke volume index (Stott *et al.* 2016*b*). However, this study included only 50 women, and most women of Black ethnic backgrounds (70%) still had an adequate response to labetalol, demonstrating that individual variation is likely to outweigh variation between ethnic groups. The same research team showed that, in 120 pregnant hypertensive women prescribed labetalol monotherapy (*n* = 120), achievement of BP control (defined as BP < 140/90 mmHg) was almost 20% lower in women of Black versus White ethnic backgrounds, although medication adherence was not explored (Stott *et al.* 2016*a*).

### Clinical use of ethnicity summary

Maternal ethnicity and/or racial origin are currently included as a clinical risk factor to guide aspirin prophylaxis in American HDP guidelines and the FMF algorithm for pre-eclampsia. No HDP guidelines endorse ethnicity or race-based prescribing; however, clinicians are likely to be influenced by out-of-pregnancy recommendations, which include stratification of antihypertensive agent choice on the basis of ethnic/racial groupings. The use of self-identified or assigned ethnic group as a reliable proxy for heritable or ‘biological’ risk is problematic. Future studies should aim to disentangle what maternal ethnicity is acting as a proxy for in the context of HDP, and whether this should influence pre-eclampsia prediction and antihypertensive prescribing.

## Viewpoint: where should we focus to improve ethnic disparities in hypertensive disorders of pregnancy?

The aetiology underlying ethnic disparities in HDP is likely to be complex and multi-factorial. Research and interventions aimed at tackling disparities should be developed and implemented with input from the communities they aim to serve, centring the voices and experiences of those affected by these disparities (Graves *et al.* 2024). A plan of action to tackle ethnic disparities in HDP from the Preeclampsia Foundation’s Racial Disparities Task Force was published in 2024, with recommendations across research, healthcare practices and community domains (Graves *et al.* 2024). Here, we summarise key actions based on our literature review across these domains.

### Research and representative data

Standardisation in collection and reporting of ethnicity data across geographic settings will be essential in confirming epidemiological trends and measuring progress. HDP studies must be inclusive and report on diverse populations, including appropriate representation of ethnic minority groups in clinical trials, biomarker studies, and genomic research, to ensure research findings are generalisable and translatable across individuals and populations. Key to succeeding in this aim, women of minoritised ethnic backgrounds need to be engaged as key stakeholders throughout the research lifecycle. Steps are being taken to improve the representation of ethnic minority groups in genomic research in the UK with government-led programmes such as the Genomics England Diverse Data initiative (Diverse 2025 Data initiative).

Further research is required to understand what ethnicity is acting as a proxy for in the context of HDP and determine the most equitable approaches to determine pre-eclampsia risk. Validation of pre-eclampsia diagnostic tests and prediction models across different ethnic groups and settings is needed. Furthermore, prospective or electronic health record cohorts with detailed social, environmental, and clinical data and associated biological samples would enable analytical approaches to understand the combined influences of biological, behavioural, environmental, socio-cultural and healthcare system factors on HDP risk and outcomes across ethnic groups, as outlined in the US National Institutes of Minority Health and Health Disparities Research Disparities framework (Alvidrez *et al.* 2019).

### Clinical care

Without definitive understanding of the drivers of ethnic disparities in HDP, it is difficult to make evidence-based recommendations on which actions are likely to improve these disparities. However, given the importance of BP control in determining maternal–fetal outcomes in HDP (Magee *et al.* 2015, Chappell *et al.* 2021), and the finding from the CHAPS trial that lower BP treatment initiation thresholds in women with chronic hypertension lowers risk of progression to pre-eclampsia (Tita *et al.* 2022), strategies targeting optimisation of BP control in women of ethnic minority backgrounds are likely to be beneficial. This is particularly salient given the high burden of pre-existing chronic hypertension in women of Black ethnic backgrounds (see section titled 'Epidemiology of hypertensive disorders').

To achieve optimal BP control, it is likely that innovative care models that extend beyond traditional clinical settings will be needed, particularly given potential mistrust in healthcare systems and barriers to accessing care in some settings (e.g. insurance status in the USA) (Suresh *et al.* 2022). Partnerships with trusted community organisations to equip community health workers and/or community spaces with education and resources for BP monitoring warrants exploration (Graves *et al.* 2024) and digital health technologies to empower women to safely monitor their BP from home with the possibility of expediting detection of hypertension, self-management of hypertension and provision of educational content (Kern-Goldberger & Hirshberg 2021). A small study demonstrated a postpartum remote BP monitoring program that resulted in a 50% reduction in Black-White disparity in postpartum BP ascertainment (RR 0.51, 95% confidence interval, 0.33–0.78) (Hirshberg *et al.* 2019). Further investigation of the role of telemedicine in antenatal and postnatal BP management across maternal ethnic groups is warranted (Graves *et al.* 2024), with several postnatal trials funded or ongoing (SNAP-2 (ISRCTN11042045), PCORI (Self-Monitoring and Responsive Technology for Postpartum Blood Pressure Control 2024).

Cultural competence and implicit bias training for healthcare professionals and wider institutional measures to tackle racism within healthcare organisations, whilst not specific to HDP, is also likely to be important (Johnson & Louis 2022). Quality metrics that assess equity and quality in hypertension care in relation to ethnic disparities, alongside frameworks for accountability in reducing disparities, will also be needed in order to identify areas for improvement and monitor effectiveness of interventions (Graves *et al.* 2024).

### Community engagement and education

Success in reducing ethnic disparities will also require meaningful engagement with communities and comprehensive education efforts (Graves *et al.* 2024). Partnerships with community organizations for education and outreach may help build trust between healthcare systems and communities. Support for patient self-advocacy and shared decision-making may be effective. Implementation of the NICE hypertension in pregnancy shared decision-making tool led to reduction in decisional conflict regarding taking antihypertensive agent medication in 50 pregnant women with chronic hypertension, with half of women in the study being of Black and Asian ethnic backgrounds (Whybrow *et al.* 2022).

## Conclusion

Despite the challenges in defining and understanding ethnicity, it is clear that ethnic disparities in HDP represent a complex global health problem, with women of Black ethnic backgrounds consistently experiencing higher rates of pre-eclampsia, HDP and associated worse outcomes across diverse healthcare settings. While traditional cardiovascular risk factors and SES contribute to these disparities, they do not explain observed differences. Research examining the complex interactions across biological, behavioural, environmental, socio-cultural and healthcare system factors on HDP risk and outcomes across maternal ethnic groups is needed to understand drivers of disease and ensure equity in pre-eclampsia prediction models and biomarker performance. Furthermore, researchers and institutions must ensure appropriate diversity and representation of ethnic minority groups in HDP research to ensure the incoming benefits of genomic and personalised medicine are applicable to women of all ethnic backgrounds, engaging minoritised women who have experienced HDP as partners and key stakeholders throughout the research lifecycle.

While the fundamental drivers of ethnic disparities in HDP remain to be fully understood, there are clear paths forward for immediate action within healthcare systems. Key priorities should include optimising BP control during pregnancy and postpartum for women from minoritised ethnic backgrounds. Critical to success is ensuring that women with lived experience are equal partners in designing and implementing these initiatives.

## Declaration of interest

FCR receives salary as the Chief Medical Officer at MEGI Health UK Ltd, holds equity in Nexus Connected Limited, where he serves as an advisor, and receives consulting fees for advisory services provided through Option 5 Health, Revena Limited and Gerson Lehrman Group Limited (GLG). The other authors have no conflicts of interest to declare.

## Funding

FCR is supported by the Medical Research Councilhttps://doi.org/10.13039/501100000265 (MR/V006835/1, external peer review). AdM is supported by the Fetal Medicine Foundationhttps://doi.org/10.13039/501100003123 (495237). LCC is supported by an NIHR Senior Investigator Award.

## Author contribution statement

FCR and LCC conceived the review. FCR drafted the first version of the manuscript, which was revised and approved by all the authors.
